# Detection of Antibodies and Confirmation of *Mycobacterium avium* Subspecies *paratuberculosis* Using Nested PCR in Bulk Milk Samples from Nakasongola and Sembabule Districts, Uganda

**DOI:** 10.1155/2013/369730

**Published:** 2013-12-16

**Authors:** Julius Boniface Okuni, Tony Oyo, Magid Kisekka, Sylvester Ochwo, David Kalenzi Atuhaire, Mathias Afayoa, William Olaho-Mukani, Lonzy Ojok

**Affiliations:** ^1^College of Veterinary Medicine, Animal Resources and Biosecurity, Makerere University, P.O. Box 7062, Kampala, Uganda; ^2^National Agricultural Research Organization, National Livestock Resources Research Institute, P.O. Box 96, Tororo, Uganda; ^3^African Union Inter African Bureau for Animal Resources, P.O. Box 30786, Nairobi 254, Kenya

## Abstract

*Mycobacterium avium* subspecies *paratuberculosis* (MAP) is an emerging pathogen in many livestock and wildlife populations around the world. Concerns range from the serious economic impacts on livestock productivity to its suspected role in the human inflammatory bowel disease syndrome. Milk and faeces of infected animals are the main vehicles through which the organism spreads from infected to susceptible hosts. In this study, a survey was done in Nakasongola and Sembabule districts of Uganda involving a total of seven dairy collection centres to determine the prevalence of antibodies to MAP in bulk milk samples. The milk was tested with a commercial ELISA kit for MAP testing in milk. Positive and suspicious milk samples were further tested using nested PCR. Of the 257 milk samples tested, 11 (4.3%) were positive and five (1.9%) were suspicious. All the ELISA positive and suspicious milk samples were positive using nested PCR. The results show that MAP infection occurs in cattle from the two districts and highlight the need for a paratuberculosis control program in these and other districts where MAP infection has been reported.

## 1. Introduction

*Mycobacterium avium *subspecies* paratuberculosis* (MAP) causes Johne's disease or paratuberculosis in ruminants and some nonruminants [1]. Infected animals develop the disease over a period of one to several years [1–3]. During the course of time the animal begins to shed the organism in its faeces and milk which enables transmission to susceptible animals through ingestion of contaminated milk or fodder [2]. Paratuberculosis is a great concern in dairy cattle and other farmed ruminants especially under intensive systems, in which it leads to deaths, early culling, and production losses, among others [2]. Prevalence of MAP in infected cattle has been determined by serological testing of serum and milk; culture of the organism from faeces and tissues; and histopathology and PCR on tissues, faeces, and milk [1]. Initial studies involving milk were primarily for determination of prevalence of infection in cattle, but focus has now turned to the public health significance of MAP shedding into the milk [4].

There have been ongoing debates on the role of MAP in the aetiology of Crohn's disease of man [5–9]. Although the causal effects of MAP on Crohn's disease are not proven beyond doubt, the suspicion has led to concerns about the zoonotic potential of MAP and that MAP should be considered one of the food borne pathogens [10, 11]. Several reports indicate that MAP can survive pasteurisation [12, 13]. This means that contaminated milk may pose risks of infection to susceptible hosts even when milk is pasteurised [4, 14]. In many developing countries such as Uganda, most of the milk is consumed without pasteurisation either after short time boiling (5–10 minutes) or as raw milk. In some countries such as India where MAP infection in livestock is widespread, MAP has been readily isolated from the human population with and without Crohn's disease, implying that constant exposure may increase the risk of infection and persistence of the organism in the blood stream of exposed people [6, 15]. MAP is not the only pathogen to be concerned about in nonpasteurised milk, but there would be legitimate concerns about consuming milk which contains live MAP bacilli given the current controversies [12, 16, 17]. Like other nontuberculous organisms, MAP could become pathogenic in immunocompromised individuals [18].

Infection of cattle with MAP in Uganda has been confirmed recently [19]. Seroprevalence of MAP infection has been reported in a few districts in central Uganda, but no countrywide study has been done on the prevalence of MAP infection in cattle and other livestock [20, 21]. There also has never been any investigation on the presence of MAP bacilli in milk marketed around the country.

Nakasongola and Sembabule districts are two of the predominantly cattle keeping districts in Uganda. The cattle comprise both those under communal grazing as well as those on private farms and ranches. These districts supply both milk and slaughter animals to the urban populations around the country; therefore the health of its livestock population and the safety of its livestock products are of paramount importance to the country.

The purpose of the study was to determine the occurrence of MAP antibodies in bulk milk supplied to urban centres from the two districts and assess if milk with antibodies to MAP may in turn contain MAP.

## 2. Materials and Methods

### 2.1. Milk ELISA

Seven established dairy collecting centres were visited in December 2012 and February 2013. Five millilitres of milk was sampled from each milk can of milk brought to the centre. Two hundred fifty-seven samples were obtained from the seven dairy collecting centres: 64 from the two centres in Nakasongola district and 193 from the five centres in Sembabule district.

No information was available on the farm or village of origin of most of the milk samples since the milk was collected from different farms and pooled into cans before being transported to dairy centres. The samples were placed in a cool box and later transported to the laboratory where they were frozen until tested. Testing of the milk samples was done using Pourquier paratuberculosis ELISA (IDEXX Montpellier SAS, France) according to the manufacturer's instruction. Briefly the samples were diluted 1 : 2 times with a dilution buffer and incubated for 15 minutes at room temperature (24–26°C) (RT); then 100 *μ*L of each sample and the positive and negative controls were dispensed into separate wells. The plate was then incubated at RT with gentle shaking on an orbital shaker. The plate was washed thrice with 250 *μ*L of the wash solution. 100 *μ*L of diluted conjugate (1 : 99) was added to the plates and incubated at RT for 30 minutes. The plate was again washed as before. 100 *μ*L of TMB substrate solution was added to each well and then incubated at RT for 10 minutes in darkness. The reaction was then stopped by adding 100 *μ*L of stop solution. The plate was read at 450 nanometres on a Biotek plate reader. Sample to positive ratio of each sample was calculated as follows: Sample_A450_-NC_A450_/PCx-NC_A450_, where Sample_A450_, NC_A450_, and PCx are the optical densities of the sample, the negative control, and the mean of the positive control, respectively, read at 450 nanometres. The manufacturer states that, for milk ELISA, a sample to positive ratio (or percentage) of <20% is considered as negative, while ≥20–<30% is suspect and ≥30% is positive. The numbers of negative, suspicious, and positive samples were thus determined.

### 2.2. DNA Extraction from the Milk

The milk was thawed in a water bath and vortexed. One millilitre of each sample was transferred to 1.5 mL microcentrifuge tube. The tubes were centrifuged at 6000 ×g for 5 min. The supernatant was discarded leaving the pellet and the fat portion. One millilitre of PBS (137 mM NaCl, 2.7 mM KCl, 10 mM Na_2_HPO_4_, and 2 mM KH_2_PO_4_, pH 7.4) was added to the sample, vortexed, and then centrifuged as done previously. The supernatant was discarded. An equal volume of 1% NaOH was added to samples, vortexed, and centrifuged. The supernatant was discarded. To each tube, 180 *μ*L of mycobacterial lysis buffer (400 mM NaCl; 40 mM Tris-HCl; 2 mM EDTA; 1.2% Triton X-100; and 0.6% SDS) was added, then vortexed, and incubated at 80°C for 15 min. DNA extraction was then carried out using GeneJET Genomic DNA extraction kit (Fermentas) following the protocol for Gram-positive bacteria. The eluted DNA was stored at −20°C.

### 2.3. Nested PCR on the Milk

PCR was done in a two-step nested protocol. The initial or primary PCR was done using the primers Map1F (5′-CAT CGG AAC GTC GGC TGG TCA GG-3′) and Map1R (5′-GAT CGC CTT GCT CAT GGC TGC CG-3′) [22]. The reaction mix consisted of 12.5 mL of 2x ready mix, 0.5 mL of 10 mM forward and reverse primers each, 6.5 mL of PCR grade water, and 5 mL of DNA template. The thermal profiles included initial denaturation of 94°C for 3 minutes and then 35 cycles with denaturation at 94°C for 60 seconds, annealing at 58°C for 60 seconds, extension at 72°C for 60 seconds, and final extension at 72°C for 3 minutes. The secondary PCR was done using primers Map2F (5′-GCA GCT CGA CTG CGA TGT CAT CG-3′) and Map2R (5′-GGC ACG GGC TGC TTT ATA TTC CC-3′) [22]. The reaction mixture consisted of 12.5 mL of 2x ready mix, 0.5 mL of forward and reverse primers, 10.5 mL of PCR water, and 1 mL of amplicons from the first PCR. The thermal conditions for the secondary PCR were similar to the primary except for the annealing temperature which was 50°C. PCR products were electrophoresed on 1% agarose gel stained with ethidium bromide and viewed under UV light.

## 3. Results and Discussion

**Figure 1:**
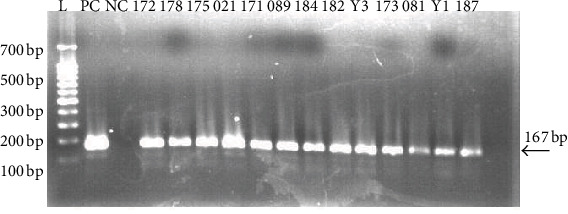
Agarose gel photograph of PCR products from amplification of MAP specific IS900 locus using nested PCR. Lane L is the 100 bp DNA ladder (New England Biolabs), lane 1 is the positive control (MAP strain K10), lane 2 is the negative control, and lanes 3–15 were milk samples 172, 178, 175, 021, 171, 089, 184, 182, Y3, 173, 081, Y1, and 187.

Of the 257 milk samples, 11 (4.3%) were positive by the ELISA, five were suspicious, while 246 were negative. The percentage of positive milk samples from Nakasongola district was 3.1% (2/64) while the percentage of positive samples in Sembabule was 4.7% (9/193) with 2.6% (5/193) as suspicious. When the primary PCR was done on the 11 ELISA positive and 5 suspicious samples, only 4 of the ELISA positives were found to be positive giving an amplicon of 217 bp. When secondary PCR was followed, all the 16 samples (11 positive and five suspicious samples) were positive showing a product size of 167 base pairs (Figure 1).

This is the first study involving testing of milk samples for paratuberculosis in the country. The finding that 4.3% of the milk contained antibodies to MAP using ELISA is close to figures in the previous seroprevalence studies from other districts in Uganda [20, 21] and the findings of a histopathology study in cattle from two abattoirs in Kampala [23], though those studies involved sera and histopathology and are not really comparable. In the present study, it is difficult to estimate the number of infected cattle since the milk was pooled before being brought to the collection centres and milk from one cow can contaminate a whole can, while the same can may contain milk from several herds. It was difficult therefore to know precisely which farms had the cows which were shedding the antibodies. The detection of MAP in these districts adds to the number of districts in Uganda with MAP infection in cattle and shows that MAP infection is more widespread in the country than was previously thought. The cattle that are shedding the organism in the milk are presumably also shedding the organism in their faeces and contaminate the pasture. Similarly, they are infecting their offspring through their milk. Thus there is an insidious spread of the infection in these two districts which had not previously been known. Nakasongola appears to have a lower rate of infection compared to Sembabule, but the number of samples was equally lower in Nakasongola due to fact that most of the milk in the district is sold to informal markets which could not be incorporated in this study. To get the actual level of MAP infection in the districts, it will be necessary to carry out herd-level surveys on sera and milk, but this study offers us a preview of the status of the districts with regard to paratuberculosis infection.

Although ELISA detected 11 positive samples and five suspicious ones, only four were detectable using the first PCR, highlighting the relative sensitivity of the ELISA. However, nested PCR was able to detect all the positive and suspicious samples as positives. The results also show that the cut-off values of Pourquier milk ELISA for paratuberculosis are within detection limits of positive milk samples. Therefore the ELISA is very robust. The confirmation of the presence of MAP DNA using nested PCR suggests that MAP is shed in milk and this could have implications to those who consume raw unpasteurised milk or milk prepared by short time boiling of 5–10 minutes [11]. Although MAP has not been proved to be a zoonotic pathogen, there are reports incriminating it in Crohn's disease [5, 24]. There is also another report in which MAP was diagnosed in an HIV infected person in Germany [18], while, in India and USA, MAP has been isolated in healthy and Crohn's disease patients [15, 25]. The case of MAP in HIV infected person speaks loud in a country like Uganda with high rate of HIV infection. This situation coupled with the culture of consuming unprocessed milk could expose susceptible individuals to MAP infection, whose outcome cannot be predicted. Though contamination of the milk by the bacteria can be deduced from this study, we could not confirm if the organisms were living and thus assess the level of risk. Also, the milk brought to the dairy centres represents a small fraction of the milk produced in the two districts; therefore no generalised conclusions about the contamination of the milk in the two districts can be made. From a “One health” perspective [26], control of paratuberculosis in livestock as well as adequate preparation of the milk to kill the highly heat tolerant MAP bacilli shall be necessary to ensure human health in this regard.

## 4. Conclusion

MAP infection is present in cattle from Nakasongola and Sembabule districts and they shed the organism in the milk which could lead to spread of the organism within herds and may pose a public health risk to people who consume unprocessed milk.
